# Immunophenotypic Characteristics of Bone Marrow Microenvironment Cellular Composition at the Biochemical Progression of Multiple Myeloma

**DOI:** 10.3390/jcm11133722

**Published:** 2022-06-27

**Authors:** Agnieszka Krzywdzińska, Bartosz Puła, Donata Szymczak, Aneta Milanowska, Agnieszka Szeremet, Krzysztof Jamroziak

**Affiliations:** 1 Laboratory of Immunophenotyping, Institute of Hematology and Transfusion Medicine, Indiry Gandhi 14, 02-776 Warsaw, Poland; 2 Department of Hematology, Institute of Hematology and Transfusion Medicine, Indiry Gandhi 14, 02-776 Warsaw, Poland; bpula@ihit.waw.pl; 3 Department and Clinic of Haematology, Blood Neoplasms and Bone Marrow Transplantation, Wroclaw Medical University, Pasteura 4, 50-367 Wroclaw, Poland; donata113@poczta.fm (D.S.); agnieszka.szeremet@wp.pl (A.S.); 4 Flow Cytometry and Cytomorphology Laboratory, Department and Clinic of Haematology, Blood Neoplasm and Bone Marrow Transplantation, University Hospital in Wroclaw, Pasteura 4, 50-367 Wroclaw, Poland; amilanowska@usk.wroc.pl; 5 Department of Hematology, Transplantation and Internal Medicine, Medical University of Warsaw, Banacha 1a, 02-097 Warsaw, Poland; kjamroziak@wum.edu.pl

**Keywords:** multiple myeloma, biochemical relapse, immune profiling, microenvironment

## Abstract

Multiple myeloma (MM) relapses are inevitable in the majority of patients, and in addition to genetic changes in the MM clone, the immune profile of the bone marrow (BM) plays a key role in this process. Biochemical progression or relapse (BR) precedes clinical relapse in a significant proportion of patients with MM. In the present study, we used flow cytometry to assess the cellular composition of the BM microenvironment in MM patients with confirmed BR. Fifteen distinct cells subsets in the BM were evaluated with the panel of antibodies used routinely for MRD monitoring in MM in 52 patients with MM (MRD-negative *n* = 20, BR *n* = 20, and clinically relapsed MM, RMM *n* = 12). The median percentage of MM cells detected in BR patients was 0.90% versus not detectable in MRD-negative patients and of 3.0% in RMM cohort. Compared to the MRD-negative group, BR status was associated with an increase in the percentage of lymphoid subpopulations, including memory B cells (*p* = 0.003), CD27+T cells (*p* = 0.002), and NK/NKT cells (*p* < 0.001). Moreover, a decrease in B-cell precursors (*p* < 0.001) and neutrophils (*p* = 0.006) was observed. There were no significant differences in the composition of the BM cell subpopulations between the BR and RMM groups. Our results indicate the involvement of B-, T-, and NK cells in the process of losing immune surveillance over the MM clone that leads to relapse. It can be speculated that similar studies of a larger cohort of BR patients can potentially identify a group of patients for which an early treatment intervention would be beneficial.

## 1. Introduction

Multiple myeloma (MM) is a hematologic neoplasm characterized by clonal proliferation of plasma cells (PCs), located primarily in the bone marrow (BM) [1]. The pathogenesis of MM is a multi-step process in which the immune system plays a critical role. Neoplastic cells characterized by genomic changes (e.g., IgH translocations or hyperdiploidy) colonize and modify the BM microenvironment to promote tumor growth. Through a wide variety of mechanisms, such as deregulation of metabolic pathways, cytokine production, and interactions with cellular and non-cellular components of BM niches, MM cells gradually create an immunosuppressive environment suitable for proliferation, survival, and drug resistance [2]. The goal of current therapeutic strategies is to eradicate MM cells and improve the immunological environment of the BM. Novel agents and regimens containing proteasome inhibitors (PIs), immunomodulatory drugs (IMIDs), monoclonal antibody agents (MoAbs), and autologous stem cell transplantation (ASCT) have revolutionized MM therapy due to the ability to interact with tumor microenvironment, enhance anti-tumor immune response, and induce restoration of bone marrow homeostasis [3]. In turn, modern diagnostic tools, such as next-generation sequencing (NGS) or next-generation flow cytometry (NGF), allow for highly sensitive monitoring of the response through measurable/minimal residual disease (MRD) assessment in the BM [4]. It was shown that MRD-negative status reduces risk of progression and predicts long-term survival and can potentially overcome the negative prognostic impact of poor prognostic features, including high-risk cytogenetics [5,6].

Nevertheless, relapse of MM is still inevitable in the majority of patients, including those who achieved deep remission with MRD negativity. The pattern of MM relapses is heterogeneous, and a significant proportion of patients present initially with a biochemical (paraprotein) progression in the absence of clinical symptoms. Such patients can remain free of therapy for a certain period, but they are at high risk of symptomatic progression [7]. It was shown that biochemical progression or relapse (BR) precedes clinical relapse, and the time interval from BR until the next treatment ranges from 10 to 13 months [7].

Potential causes of MM progression include inherent molecular changes in the plasma cell clone as well as alterations of the immune microenvironment. BM cellular microenvironment changes can lead to neoplastic cells escaping from immunosurveillance, which contribute to the selection and clonal evolution [8,9]. The close dependence of MM cells on the conditions of the BM microenvironment suggests that the immune status could be a significant part of complex individual patients’ prognostic profile; however, the diagnostic utility of assessing additional cellular populations remains unknown. Indeed, previous studies demonstrated a beneficial effect of the specific cellular profile in regard to predicting survival of MM patients, such as neutrophil-to-lymphocyte ratio, CD4+ and CD8+ T lymphocytes, and subpopulations of B cells, NK cells, or myeloid-derived suppressor cells (MDSCs) [10,11,12]. However, due to evaluations with different specimens, assays, and time points, it is difficult to draw unequivocal conclusions, highlighting the need for consensus guidelines and standardization in this area [13]. 

One of the methods to evaluate the BM cellular microenvironment is flow cytometry. The use of the next-generation flow cytometry (NGF) MM MRD protocol, developed by EuroFlow Consortium, detects MRD with a high sensitivity of 10^−5^–10^−6^, and its panel of antibodies enables the identification of several subpopulations of cells in BM during routine MRD assessment. The immunophenotypic characterization of leukocyte subpopulations in BM has shown to be of prognostic value in transplant-ineligible elderly patients. Paiva et al., using MM MRD panel of markers, defined a unique BM profile with elevated erythroblasts and B-cells precursors and decreased levels of memory and naïve B cells as a predictor of inferior outcome independent from patients’ MRD status [14]. Furthermore, the immunological profile of patients with different presentations of plasma cell dyscrasias or with MRD-negative and -positive status has been characterized in previous studies and thus may serve as a reference when testing for potential BM microenvironment alterations in other clinical situations [15,16]. 

To the best of our knowledge, there are limited data regarding the BM microenvironment changes in the early preclinical phase of MM relapse, such as BR. Therefore, we used NGF antibody panel to assess the level of infiltration with clonal plasma cells along with BM cellular composition in MM patients with BR and compared the results to those obtained in patients with complete remission (CR) MRD-negative (MRDneg) and patients with clinical relapse (relapsed MM, RMM).

## 2. Materials and Methods

### 2.1. Patients

A retrospective analysis of the results of immunophenotyping in BM of MM patients, obtained in the period January 2018–December 2021 as part of the standard diagnosis procedure, was performed. Bone marrow samples from a total of 52 MM patients (median age 68 years; range 42–80) treated in the Institute of Hematology and Transfusion Medicine in Warsaw and in the Department of Hematology of Wroclaw Medical University were analyzed. Regarding MM status, the studied cohort included: patients with MRD-negative CR (MRDneg group, *n* = 20), patients with biochemical relapse (BR group, *n* = 20), and patients with clinical relapse of MM (RMM group, *n* = 12). Definitions of hematologic responses and progression were derived from the International Myeloma Working Group (IMWG) criteria [17]. MRDneg group consisted of patients in CR with undetectable MRD by the NGF BM assessment with a minimum detection sensitivity of 10^−5^. BR group was defined as patients meeting at least one of the biochemical criteria of progressive disease according to IMWG response criteria, e.g., increase of >25% from the lowest response value in any one of the following:

- Serum M-component (the absolute increase must be >0.5 g/dL), and/or
- Urine M-component (the absolute increase must be >200 mg/24 h), and/or
- In patients without measurable serum and urine M-protein, the difference between involved and uninvolved FLC levels (the absolute increase must be >10 mg/dL) and not meeting any of the clinical criteria of progressive disease according to IMWG response criteria [17,18].

RMM group consisted of patients relapsing after any line of treatment who met any clinical IMWG criterion of progressive disease. The study was approved by the Bioethical Committee of the Institute of Hematology and Transfusion Medicine in Warsaw. 

### 2.2. Flow Cytometry Assessment of Plasma Cells and BM Microenvironment

The samples were tested in two flow cytometry laboratories with previously confirmed compliance of protocols and obtained results in MM MRD assessment [19]. EDTA-anticoagulated BM samples (*n* = 52) were processed within 24 h after collection, and following the NGF lyse-wash-stain, sample standard preparation protocol was used, as previously described [4,20]. Briefly, to monitor MRD, bulk-lysed BM samples were stained with the panel of antibodies proposed by EuroFlow. It was comprised of two 8-color tubes containing the following antibodies: 1st tube—CD27–BV510 (clone O323, BioLegend, San Diego, CA, USA ), CD138–BV421 (MI15, BD Biosciences, San Jose, CA, USA), CD38–FITC (Multi-epitope CYT–38F2, Cytognos, Salamanca, Spain), CD56–PE (C5.9, Cytognos, Salamanca, Spain), CD45–PerCPCy5.5 (HI30, BioLegend, San Diego, CA, USA), CD19–PE-Cy7 (J3-119, Beckman Coulter, Brea, CA, USA), CD117–APC (104D2, BD Biosciences, San Jose, CA, USA), CD81–APC-H7 (M38, Cytognos, Salamanca, Spain); 2nd tube—where instead of CD117 and CD81, polyclonal kappa–APC (Dako, Agilent, Santa Clara, CA, USA) and polyclonal lambda–APC-H7 (Cytognos, Salamanca, Spain) were used. For the detection of cytoplasmic antigens (kappa and lambda light chains), appropriate fixation and permeabilization steps were performed after staining for the cell membrane markers, using the Fix&Perm (Invitrogen, Waltham, MA, USA) or IntraPrep (Beckman Coulter, Brea, CA, USA) permeabilization reagent. The cut-off for MRD-negativity level was defined as <20 clonal plasma cells out of at least 2 million nuclear cells (minimal sensitivity of 10^−5^). The limit of detection (LOD) achieved in the assay was determined in each sample according to the formula (20/nucleated cells) × 100. Patients were considered to have undetectable MRD when phenotypically aberrant clonal PCs were either absent or present at percentages below the LOD achieved in the corresponding sample. BD FACSCantoII instruments (BD Bioscience, San Jose, CA, USA) were used for samples acquisition. Analysis of cytometric data files was conducted with Infinicyt (Cytognos S.L., Salamanca, Spain) and with BD FACS Diva 6.1 (BD Bioscience, San Jose, CA, USA) in MRD assessment and immune profiling, respectively.

**Figure 1:**
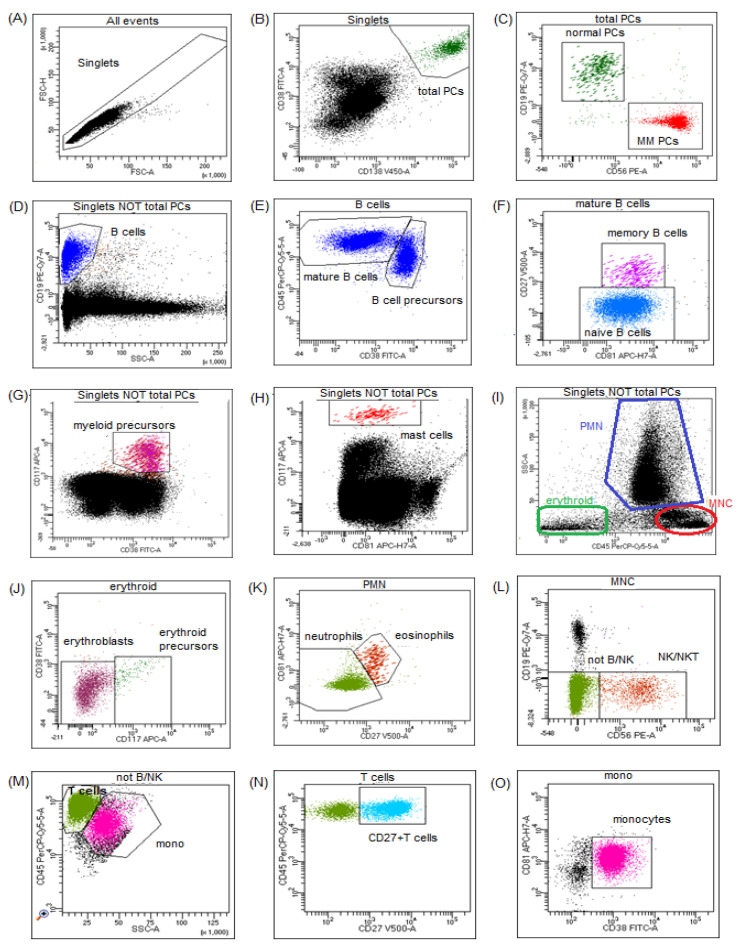
Gating strategy used for identification of the different populations of nuclear cells in BM from a representative patient in biochemical relapse of MM. (**A)** Determination of nucleated cells population by excluding doublets on FSC−H/FSC−A dot plot—gate singlets. (**B**) Total PCs population was determined by gating events CD38+highCD138+. (**C**) MM PCs (in red) were distinguished from normal PCs (in green) by aberrant immunophenotype: CD19−CD56+bright. (**D**) On the dot plot showing nuclear cells without the PCs population, B−cells region CD19 + SSClow was drawn. (**E**) On bivariate plot CD45 versus CD38 gated on B−cells regions defining mature B cells and B cell precursors were drawn. In the next graph (**F**) showing only mature B−cells, naive and memory B−cells were distinguished based on CD27 and CD81 expression. On bivariate plots gated on singlets and not PCs, myeloid precursors CD38+CD117+ (**G**) and mast cells CD117+bright (**H**) were identified. Among nucleated cells on the bivariate plot of CD45 versus SSC−A (**I**), population of erythroid CD45−/dim, polymorphonuclear cells (PMN), and mononuclear cells (MNC) were gated. On plot CD38 versus CD117, gated on erythroid cells erythroblasts and erythroid precursors CD117+ were identified (**J**). Population of PMN was divided on neutrophils and eosinophils based on the expression of CD81 and CD27 (**K**). In population of MNC, the lymphoid cells that express CD56 antigen were identified as NK/NKT cells (**L**). The rest of events without CD19 and CD56 expression and with lymphoid characteristic on CD45 versus SSC−A dot plot were classified as T cells (**M**). Next, a subpopulation of T cells with positive expression of CD27 was gated (**N**). Finally, on the bivariate plot of CD38 versus CD81, population of monocytes was precisely distinguished among CD45+SSCint nuclear cells (**O**). BM, bone marrow; MM, multiple myeloma; PCs, plasma cells.

The gating strategy used in the analysis is depicted in Figure 1. 

Neoplastic PCs were identified by gating CD138+ CD38+^high^ cells that express aberrantly specific markers (CD56, CD19, CD45, CD27, CD81, or CD117) and its clonal nature confirmed by the restricted expression of cytoplasmic kappa or lambda light chains. Immunophenotyping of the BM microenvironment was performed using the first eight-color combination of antibodies (CD27, CD138, CD38, CD56, CD45, CD19, CD117, CD81). Identification of 15 subpopulations of leukocytes (seven myeloid and eight lymphoid subsets) was based on the phenotypic criteria: erythroblasts (CD38− CD45− SSC^low^), erythroid precursors (CD38− CD45− CD117+ SSC^low^), monocytes (CD38+ CD45+ CD81+ SSC^int^), mast cells (CD45+^dim^ CD117+^bright^), eosinophils (CD45+^bright^ CD81+ SSC^high^), myeloid precursors (CD38+ CD45+^dim^ CD117+ SSC^high^), and neutrophils (CD45+^dim^ SSC^high^). Among the total population of lymphocytes CD45+SSC^low^, we identified: B cells (CD19+ CD45+ SSC^low^) and their subsets: transitional/naïve (CD19+ CD27− CD38−/+^dim^ CD45+), memory (CD19+ CD27+ CD38−/+^dim^ CD45+), and B-cell precursors (CD19+ CD27− CD38+^bright^ CD45+^dim^) and T cells (CD19− CD45+ CD56− SSC^low^), CD27+T cells (CD19− CD45+ CD56− CD27+ SSC^low^), and NK/NKT cells (CD19− CD45+ CD56+ SSC^low^). Normal plasma cells were identified as CD138+ CD38+^high^ CD45+/− CD19+/− CD56−/+ CD27+ CD81+ CD117−. Following previously defined criteria, the quality of the BM aspirates was assessed based on the presence of ≥0.002% mast cells CD117+^bright^ [4]. 

### 2.3. Statistical Analysis

The non-parametric Kruskal–Wallis test and post hoc analysis were used to evaluate the statistical significance of the differences between groups. Correlation studies were performed using the Spearman test. *p*-values < 0.05 were considered statistically significant. Analyses were conducted using Prism 7.0, (GraphPad Software, La Jolla, CA, USA).

## 3. Results

Clonal plasma cells and BM microenvironment composition were assessed in 52 MM patients with median age of 68 (range: 42–80), including 20 patients with BR and two reference populations: 20 patients with MRDneg and 12 patients with RMM. The clinical and laboratory patients’ characteristics are summarized in Table 1. 

**Figure 2:**
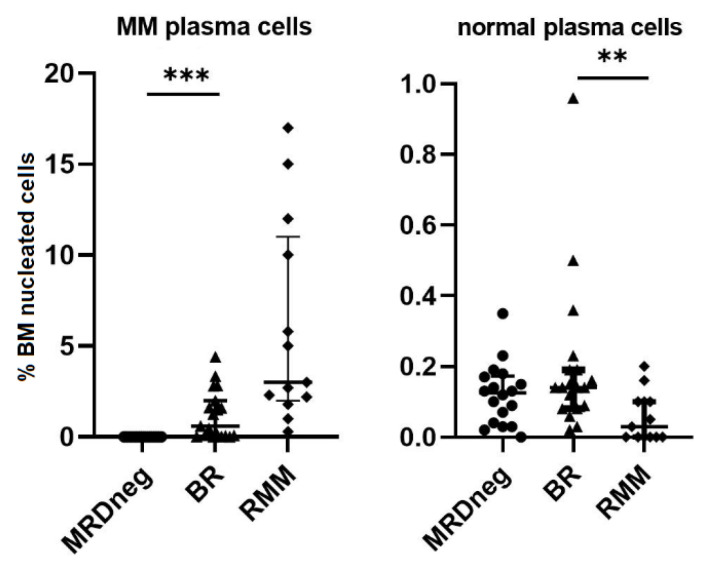
Distribution of clonal plasma cells and normal plasma cells in BM samples from MRD negative patients in biochemical relapse (BR) and clinical relapsed MM (RMM). Graphs show the median and quartile Q1–Q3 values (** *p* < 0.01; *** *p* < 0.001). BM, bone marrow; MRD, measurable residual disease.

A median of 2,900,000 nucleated BM cells per file (range: 2,250,000–5,100,000) was studied in MRD testing. In the BR group, a median of 950,000 (700,000–2,100,000) and in the RMM a median of 430,000 (200,000–970,000) nucleated BM cells were analyzed. BM samples showed no significant levels of hemodilution with median (range) frequencies of mast cells of 0.004% (0.002–0.037%). MRD negativity was achieved with a median LOD of 0.0006% (i.e., 6 × 10^−6^); therefore, for the MRDneg group, the median MRD status was determined as <0.0006%. Median percentages of clonal MM cells detected were 0.90% (0.10–4.40%) in the BR group of patients and 3.0% (0.5–17.0%) in the RMM patients cohort (Figure 2). The serum protein electrophoresis test results of 19 patients in the BR group and 10 in the RMM group showed no significant correlation between the percentage of MM cells and monoclonal protein concentration (median 7 g/L vs. 14 g/L, r = 0.42, *p* = 0.065). No monoclonal protein was detected in the MRDneg group.

**Figure 3:**
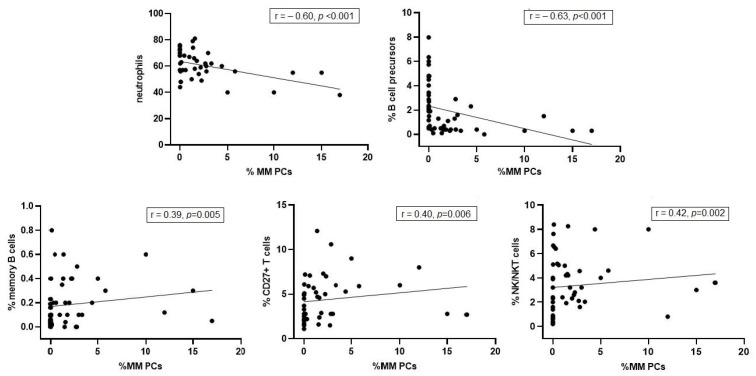
Correlation between percentage of MM plasma cells and the percentage of neutrophils, B−cell precursors, memory B cells, CD27+ T cells, and NK/NKT cells in bone marrow aspirates from MM patients: MRD negative (*n* = 20) with biochemical relapse (*n* = 20) and with clinical relapse (*n* = 12).

Analysis of correlations of MM infiltration and BM cells populations showed that the increasing number of MM cells negatively correlates with the percentage of neutrophils (r = −0.60, *p* < 0.001) and B-cells precursors (r = −0.63, *p* < 0.001). Moreover, with increasing percentage of MM PCs in bone marrow, we also found a modest increment of memory B cells (r = 0.39, *p* = 0.005), CD27+ T cells (r = 0.40, *p* = 0.006), and NK/NKT cells (r = 0.42, *p* = 0.002) (Figure 3).

**Figure 4:**
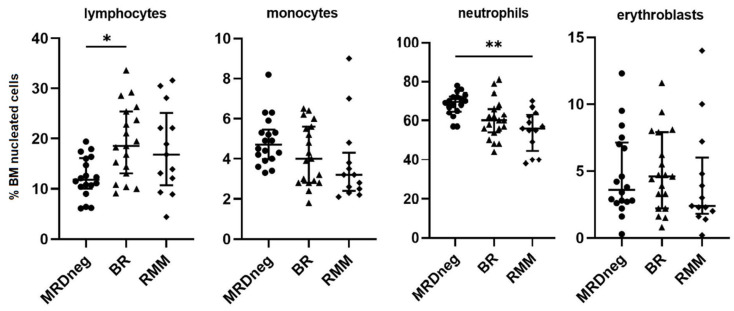
The differences in the median proportion of main BM populations: lymphocytes, monocytes, neutrophils, and erythroblasts between MRD negative patients with biochemical relapse (BR) and relapsed multiple myeloma (RMM) patients. Graphs show the median and quartile values Q1–Q3 (* *p* < 0.05; ** *p* < 0.01). BM, bone marrow; MRD, measurable residual disease.

The distribution of the main leukocyte subsets was heterogeneous among patient groups, showing the unique individual BM microenvironmental signature. The comparison of relative frequencies of the major populations of BM (total lymphocytes, monocytes, neutrophils, and erythroblasts) showed no pronounced differences between study groups, and only for neutrophils (70% in MRDneg vs. 56% in RMM, *p* = 0.006) and total population of lymphocytes (11.8% in MRDneg vs. 18.5% in BR, *p* = 0.04), the numbers of cells were significantly altered (Figure 4). There was no difference between the studied groups in relation to the frequency of other studied myeloid subpopulations: eosinophils, mast cells, myeloblasts, and erythroid progenitors.

**Figure 5:**
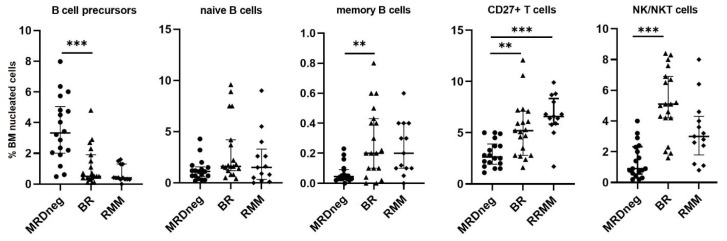
The differences in the median proportion of BM lymphocytes subsets: B−cells precursors, naïve and memory B cells, CD27+T cells, and NK/NKT cells in MRD negative patients with biochemical relapse (BR) and relapsed multiple myeloma (RMM) patients. Graphs show the median and quartile Q1–Q3 values. (** *p* < 0.01; *** *p* < 0.001). BM, bone marrow; MRD, measurable residual disease.

MM progression had no effect on the relative percentage of the total B−cell population (median total B cells 4.7%, 3.0%, and 3.1% in MRDneg, BR, and in RMM, respectively). However, more detailed assessment of the B-cells compartment showed a significant reduction of B-cell precursors in the BR and RMM groups (median 3.3%, 0.54%, and 0.40% in MRDneg, BR, and RMM, respectively; *p* < 0.001 for MRDneg vs. BR). In mature lymphocytes B cluster, a significantly higher percentage of memory B cells CD19+CD27+ in BR patients was observed (median 0.045% in MRDneg vs. 0.16% in BR, *p* = 0.003), whereas the number of transitional/naïve B cells was comparable (median 1.1%, 1.6%, and 1.5% in MRDneg, BR, and in RMM, respectively). The increase in BM infiltration with MM cells corresponded with a marked decrease in the number of normal PCs only in RMM group (median 0.13%, 0.12%, and 0.05% for MRDneg, BR, and RMM, respectively; *p* = 0.002 for BR vs. RMM) (Figure 5).

Interestingly, a gradual increase in the T-cells population was noticed from MRDneg remission through BR to clinical relapse (median T cells 4.6%, 7.6%, and 11.0% for MRDneg, BR, and RMM, respectively; *p* = 0.02 for MRDneg vs. RMM). Proportion of T cells with CD27 antigen expression was higher in both relapsed groups (median T cells CD27+ 2.7%, 5.2%, and 6.1% for MRDneg, BR, and RMM, respectively; *p* = 0.002 for MRDneg vs. BR, *p* < 0.001 for MRDneg vs. RMM), whereas no differences were seen regarding the CD27-negative T cells (*p* = 0.5). Similarly, NK/NKT cells enhanced their contribution to the lymphoid cell population (median 0.9%, 5.1%, and 3.0% for MRDneg, BR, and RMM, respectively; *p* < 0.001 for MRDneg vs. BR). 

## 4. Discussion

Biochemical relapse (BR) is a clinical situation when biochemical (paraprotein) criteria of progressive disease are met in the absence of clinical symptoms. Patients with BR are at high risk of developing symptomatic progression. It was shown that BR precedes clinical relapse, with a time interval from BR until the next treatment from 10 to 13 months [7]. However, the current recommendation of the IMWG for patients with relapsed/refractory MM and signs of progression is not to treat until they develop clinical relapse (CRAB symptoms, development of new plasmacytomas or definite increase of 50% of existing plasmacytomas or bone lesions, or hyperviscosity requiring therapeutic intervention) except in those who developed “significant paraprotein relapse” [18]. Significant paraprotein relapse is defined as doubling of the M-component in two consecutive measurements separated by <2 months or an increase in the absolute levels of serum M protein by 1 g/dL, urine M protein by 500 mg/24 h, or involved serum FLC level by 20 mg/dL (plus an abnormal FLC ratio) in two consecutive measurements separated by <2 months [18]. 

In the present study, we assessed BM infiltration with clonal plasma cells as well as BM microenvironment cellular composition by NGF method in patients with BR of MM and compared it to patients with MRDneg status as a “pre-relapse” group and patients with confirmed clinical relapse (RMM group). The NGF MM MRD panel of antibodies provides an opportunity for generating individualized BM niche profiles for each patient and hence to evaluate whether the stadium of MM correlates with differences in the relative distribution of the BM subset of cells. The increase of monoclonal protein observed in BR patients is likely a result of increased proliferation of neoplastic plasma cells accumulating in the bone marrow. In line with this, median MM PCs detected in BM aspirates of BR patients was 0.90% versus not detectable MM PCs in MRDneg and median of 3.0% in RMM cohort. Although the differences between BR and RMM groups were not statistically significant (*p* = 0.07), the similar percentage of normal PCs in MRDneg and BR patients and significantly lower one in RMM (*p* = 0.002) indicate that the BR group represents an early stage of MM recurrence. 

In our study, the BM immune profile of MRDneg patients in CR was assessed after the first treatment line with ASCT consolidation, at the interval of 100–180 days after the transplantation procedure. This does not fully correspond to the profile of healthy donors due to the increased or often incomplete regeneration after transplantation, but it may to some extent reflect the immunological status of the control over neoplastic cells [21]. However, in most patients, post-ASCT state of the myeloma-immune equilibrium is followed by immunological escape and subsequent progression, facilitated by multiple factors including alternations in cytokine production, T-cells exhaustion, and accumulation of macrophages protecting MM cells from apoptosis [22,23]. Our analysis may imply that loss of tumor cell surveillance at the stage of asymptomatic BR is associated with an increase in the percentage of lymphoid subpopulations including CD27+ T cells, memory B cells, and NK/NKT cells. Furthermore, marked decrease in B-cell precursors and neutrophils was observed in BM during the course of the disease. At the same time, we found no significant differences in the composition of the analyzed BM cell subpopulations between the BR and RMM groups. We did not demonstrate any significant contribution of erythroid lineage cells, monocytes, mast cells, eosinophils, or myeloid precursors to the recurrence process.

It is likely that changes in the immune profile during MM relapse occur gradually. However, in a progression model that included smoldering multiple myeloma (SMM), MM, and plasma cell leukemia (PCL), no significant differences in relative frequencies of the major immune subsets (B cells, T cells, NK/NKT, monocytes, or erythroblasts) were detected [15]. In turn, studies comparing the immunological status between MRD-negative and MRD-positive patients showed that the presence of MRD (detected at the 10^−5^ level) is accompanied by changes within the BM, including a relative increase of erythroblasts, monocytes, memory B cells, and to a lesser extent with an increased proportion of NK and NK-T cells [15,24]. It is worth noting that the reappearance of MRD does not yet mean a recurrence. Although recent studies indicate that relapse of MM can be expected in approximately 70% of MRD-positive patients, it is known that in some of these patients, the disease can remain under control for a long time [25,26]. According to the study of Arteche-Lopez et al., patients in long-term CR express a particular immune signature with an increase in naïve B cells, reduction of memory B cells, and increment CD4+ and CD8+ effector memory T cells [26]. 

Compared with MRD-negative patients, the appearance of neoplastic plasma cells in the bone marrow in our BR cohort resulted in changes primarily in the lymphoid compartment. We did not detect differences in percentages of analyzed myeloid subpopulations or their precursors between studied groups, but populations of neutrophils were progressively lower in BR and RMM compared to MRD-negative patients. Although this may be partly due to the increase in the percentage of the MM cell population, it is worth noting that the panel of antibodies used did not allow to distinguish of specific granulocytes subpopulations. Perez et al., in two functional assays, showed a progressive gradient of immunosuppression across maturation stages of granulocytes [27]. They suggest that identification of granulocytic myeloid-derived suppressor cells (G-MDSCs) in MM patients may provide additional prognostic information. Mature neutrophils defined as CD11b+CD13+CD16+ had T-cell immunosuppressive potential, and patients with G-MDSCs/T-lymphocyte ratio higher than 3.4 had significantly inferior PFS (*p* < 0.001) [27]. 

B-cell regeneration during therapy has been considered as a strong prognostic factor in MM. More complete recovery of BM B cells and higher normal PCs component after treatment predict better outcomes in MM patients [11,28]. This was confirmed for transplant-eligible population of patients [29] as well as those with long-term disease control [26]. Changes that occurred in the composition of B-cells component in BR and RMM groups were similar and reflected competition and progressive replacement of BM niches by MM cells, which can also actively induce apoptosis of B-cell progenitors. Indeed, significant differences in frequencies of precursor B-cells between MRDneg and BR groups were observed (*p* < 0.001) and, as a consequence, increase of post-germinal memory B cells in BR patients (*p* = 0.003). Interestingly, although median proportion of normal PCs in total PCs cluster in BR patients was of 11% vs. 100% in MRDneg group, no significant differences in frequencies of normal PCs within the whole BM was seen between these groups. Significant decrease of normal PCs in RMM patients (*p* = 0.002) was observed, where median proportion of normal PCs in total PCs was 1.4%. It should be noted that we report the relative distribution of the populations as a percentage of BM where cell numbers might be influenced by the recovery or changes of other populations. Nevertheless, the change in the composition of B-cell subpopulations noted in patients in BR confirms the gradual impairment of the immune system seen in both clinical recurrence and in newly diagnosed MM patients [8,21].

Comparing the BR group with MRDneg patients, the proportion of T cells increased during disease progression. The proportion of CD27+ subpopulation was significantly higher in the BR and RMM patients compared to MRDneg group (*p* = 0.002). According to results of Botta et al., these cells may represent senescent and exhausted mostly CD4+ T- cell clusters with immune-suppressive phenotype represented by higher expression of immune checkpoint receptors, including PD-1, TIM-3, and TIGIT [30]. The studies in a subset of patients from the PETHEMA/GEM2012MENOS65 study, where immune monitoring studies were performed using 17-color NGF as well as combined scRNA/TCR sequencing, revealed that newly diagnosed MM patients with higher CD27(−)/CD27+ T- cell ratios had prolonged progression-free survival, possibly due to reactivation of CD27(−) T cells after treatment [30]. This is in line with Arteche-Lopez et al.’s hypothesis that post-ASCT patients in long-term CR may be protected from recurrence by effector memory CD27(−) T lymphocytes and higher levels of naive B cells [26]. Since we did not observe differences in frequencies of the potential MM-reactive CD27(−)T cells between studied groups of patients, this may suggest that the suppression of the anti-myeloma potential of T cells is caused by the accumulation of exhausted T lymphocytes unable to control tumor growth. 

As NK cells usually reconstitute to normal levels within one month of ASCT [31], significantly a higher percentage of NK/NKT cells in BR compared to the MRDneg group indicates their significant participation in the process of MM progression. Previous functional studies confirmed unequivocally an association between advanced disease status and a reduced cytotoxicity of NK and NKT cells [32,33]. Simultaneously, NK cells could remain functional in patients achieving long-term disease control, supporting the ability of the immune system to limit the growth of MM PCs [34]. NK cells have been shown to be especially important in the context of IMiDs, such as thalidomide, lenalidomide, and pomalidomide, since these agents stimulate IL-2 production, which promotes NK cell expansion and activation [35]. 

The limitations of our study were the relatively small sample size of enrolled patients, which limited the statistical significance of the comparisons. Moreover, we were unable to study paired samples collected from uniformly treated patients over the course of their disease. Importantly, the small sample size as well as the heterogeneity regimens of patients did not allow for subgroup analyses to determine whether the composition of the microenvironment depended on prior therapy. The most routinely used drugs, such as PI and IMIDs, act pleiotropically, exhibit different mechanisms of action, and are employed either as monotherapies or in various combinations. Although previous studies have shown that they alter the bone marrow microenvironment [15,36,37], Firer et al. pointed out the conflicting results reported that may reflect the difference between studies or different patient populations evaluated [38]. They highlighted the need to carefully design experiments that can lead toward a better understanding of the effects of treatment regimens on the anti-myeloma response [38]. It is worth noting that distribution of different subpopulation of BM cells might depend not only on prior treatment but also may differ in separate prognostic groups and may change during recovery from therapy [13,21,39]. The relapse status we referred not to healthy donors but to the MRD-negative status, which, from our point of view, is closest to the actual condition of the BM microenvironment of patients after effective treatment and which is modulated in the disease-progression process. In turn, more heavily pretreated patients have highly proliferative malignant clones that downregulate pathways required for tumor immunosurveillance [40], which limits the reliability of relating our data to healthy individuals.

The panel of antibodies used in our study did not allow for a deeper characterization of immunosuppressive cell populations, such as regulatory T cells (Treg) and tumor-associated macrophages (TAMs). Treg are characterized by CD3+CD4+CD25+ FoxP3+ immunophenotype. Although its prognostic significances is still a matter of debate, the enhanced expansion and suppressive activity of Tregs contribute to tumor cell growth, proliferation, and survival [41]. Moreover, Treg-depleting or -inhibiting therapies are under investigation to intensify antitumor immunity [42]. In turn, the anti-inflammatory M2 macrophages—tumor-associated macrophages (TAMs)—are known to be associated with the growth of MM by suppressing T-cell activity and inducing resistance to chemotherapy. Clinical studies confirmed the correlation between a higher CD163/CD68-positive M2-type macrophages infiltration of BM and a more aggressive disease and reduced survival [43]. 

The use of the EuroFlow MM MRD antibody panel provides the opportunity to assess at least 15 subsets of BM cells, which allows quantitative assessment of the immune status of each patient at different stages of disease or treatment during routine MRD evaluation. Our study for the first time provides knowledge of the immune profile changes in BM during BR preceding clinical relapse of MM, which could be observed using a routine MRD assessment method. We provided additional insights into changes in relative numbers of BM leukocytes subpopulations and suggest the involvement of B, T, and NK lymphocyte subsets in the progression course of MM. However, additional studies are needed to confirm the functional role of these subpopulations in the loss of immune surveillance of the MM clone. Recent clinical studies suggest that early use of immunomodulatory drugs, which are known to activate both innate and acquired immunity, may delay progression to clinical MM contribute to better outcomes for patients [44]. As the suitability of early treatment at the occurrence of BR is still a matter of debate, studies of the BM microenvironment composition of a larger group of BR patients can potentially identify patients at higher risk of recurrence for which earlier therapeutic intervention would be beneficial. Further research is needed to determine whether the immune profile, next to the MRD assessment, baseline risk stratification, or genetic mutations, has a chance of becoming part of the comprehensive diagnostics of MM patients. 

## Figures and Tables

**Table 1:** Clinical characteristics of MM patients groups at the time of diagnosis and the treatment received.

Parameter	CR MRDneg Group (*n* = 20)	BR Group (*n* = 20)	RMM Group (*n* = 12)
Age (years)	58 (52–70)	58 (51–69)	59 (54–67)
Gender (male/female %)	40/60	45/55	50/50
MM type at diagnosis (%)			
IgA kappa	0	1/20 (5%)	0
IgA lambda	4/20 (20%)	2/20 (10%)	5/12 (41.5%)
IgG kappa	9/20 (45%)	13/20 (65%)	3/12 (25%)
IgG lambda	5/20 (25%)	3/20 (15%)	2/12 (16.5%)
Kappa	2/20 (10%)	1/20 (5%)	1/12 (8.5%)
Lambda	0	0	1/12 (8.5%)
ISS stage at diagnosis (%)			
I	9/20 (45%)	9/20 (45%)	4/12 (33.5%)
II	7/20 (35%)	8/20 (40%)	3/12 (25%)
III	4/20 (20%)	3/20 (25%)	5/12 (41.5%)
Cytogenetics at diagnosis (%)			
High risk *	3/20 (20%)	3/20 (15%)	6/12 (50%)
Non-high risk	7/20 (50%)	12/20 (60%)	3/12 (25%)
Not available	10/20 (30%)	5/20 (25%)	3/12 (25%)
Number of previous lines of therapy, median (range)	1	1 (1–3)	2 (1–4)
Previous ASCT	20/20 (100%)	13/20 (65%)	11/12 (91.5%)
Type of previous therapy			
(%) of patient treated with			
a given regimen:			
VTD	18/20 (90%)	12/20 (60%)	6/12 (50%)
VD	1/20 (5%)	0	3/12 (25%)
PAD	1/20 (5%)	1/20 (5%)	2/12 (16.6%)
VMP	0	1/20 (5%)	0
CTD	0	6/20 (30%)	3/12 (25%)
DVD	0	1/20 (5%)	1/12 (8.3%)
Rd	0	0	3/12 (25%)
VCD	0	0	4/12 (33.3%)
KD	0	0	1/12 (8.3%)
PCD	0	0	1/12 (8.3%)

Abbreviations: ASCT, autologous stem cell transplantation; BR, biochemical relapse; CR, complete remission; ISS, international staging system; MM, multiple myeloma; MRD, measurable residual disease; RMM, relapse of multiple myeloma. VTD, bortezomib, thalidomide, dexamethasone; VD, bortezomib, dexamethasone; PAD, bortezomib, adriamycin, dexamethasone; VMP, bortezomib, melphalan, prednisone; CTD, cyclophosphamide, thalidomide, dexamethasone; DVD, daratumumab, bortezomib, dexamethasone; Rd, lenalidomide, dexamethasone; VCD, bortezomib, cyclophosphamide, dexamethasone; KD, carfilzomib, dexamethasone; PCD, pomalidomide, cyclophosphamide, dexamethasone.* Defined as the presence of t(4;14), t(14;16), t(14;20), del(17p), amp(1q).

## Data Availability

Not applicable.

## References

[1] Palumbo A., Anderson K. (2011). Multiple myeloma. N. Engl. J. Med..

[2] Leone P., Solimando A.G., Malerba E., Fasano R., Buonavoglia A., Pappagallo F., De Re V., Argentiero A., Silvestris N., Vacca A. (2020). Actors on the Scene: Immune cells in the myeloma niche. Front. Oncol..

[3] Lopes R., Ferreira B.V., Caetano J., Barahona F., Carneiro E.A., João C. (2021). Boosting immunity against multiple myeloma. Cancers.

[4] Flores-Montero J., Sanoja-Flores L., Paiva B., Puig N., García-Sánchez O., Böttcher S., van der Velden V.H.J., Pérez-Morán J.J., Vidriales M.B., García-Sanz R. (2017). Next generation flow for highly sensitive and standardized detection of minimal residual disease in multiple myeloma. Leukemia.

[5] Munshi N.C., Avet-Loiseau H., Anderson K.C., Neri P., Paiva B., Samur M., Dimopoulos M., Kulakova M., Lam A., Hashim M. (2020). Large meta-analysis establishes the role of MRD negativity in long-term survival outcomes in multiple myeloma patients. Blood Adv..

[6] Paiva B., Puig N., Cedena M.T., Rosiñol L., Cordón L., Vidriales M.B., Burgos L., Flores-Montero J., Sanoja-Flores L., Lopez-Anglada L. (2020). Measurable residual disease by Next-Generation Flow Cytometry in multiple myeloma. J. Clin. Oncol..

[7] García-Sanz R., Oriol A., Moreno M.J., de la Rubia J., Payer A.R., Hernández M.T., Palomera L., Teruel A.I., Blanchard M.J., Gironella M. (2015). Zoledronic acid as compared with observation in multiple myeloma patients at biochemical relapse: Results of the randomized AZABACHE Spanish trial. Haematologica.

[8] Paiva B., Pérez-Andrés M., Vídriales M.B., Almeida J., de las Heras N., Mateos M.V., López-Corral L., Gutiérrez N.C., Blanco J., Oriol A. (2011). Competition between clonal plasma cells and normal cells for potentially overlapping bone marrow niches is associated with a progressively altered cellular distribution in MGUS vs. myeloma. Leukemia.

[9] Busch A., Zeh D., Janzen V., Mügge L.O., Wolf D., Fingerhut L., Hahn-Ast C., Maurer O., Brossart P., von Lilienfeld-Toal M. (2014). Treatment with lenalidomide induces immunoactivating and counter-regulatory immunosuppressive changes in myeloma patients. Clin. Exp. Immunol..

[10] Zhaoyun L., Rong F. (2021). Predictive role of immune profiling for survival of multiple myeloma patients. Front. Immunol..

[11] Ho C.M., McCarthy P.L., Wallace P.K., Zhang Y., Fora A., Mellors P., Tario J.D., McCarthy B.L.S., Chen G.L., Holstein S.A. (2017). Immune signatures associated with improved progression-free and overall survival for myeloma patients treated with AHSCT. Blood Adv..

[12] Görgün G.T., Whitehill G., Anderson J.L., Hideshima T., Maguire C., Laubach J., Raje N., Munshi N.C., Richardson P.G., Anderson K.C. (2013). Tumor-promoting immune-suppressive myeloid-derived suppressor cells in the multiple myeloma microenvironment in humans. Blood.

[13] Holstein S.A., Ye J.C., Howard A., Bhutani M., Gormley N., Hahn T., Hillengass J., Krishnan A., Landgren C.O., Munshi N.C. (2019). Summary of the Second Annual BMT CTN Myeloma Intergroup Workshop on minimal residual disease and immune profiling. Biol. Blood Marrow Transplant..

[14] Paiva B., Cedena M.T., Puig N., Arana P., Vidriales M.B., Cordon L., Flores-Montero J., Gutierrez N.C., Martín-Ramos M.L., Martinez-Lopez J. (2016). Minimal residual disease monitoring and immune profiling in multiple myeloma in elderly patients. Blood.

[15] Papadimitriou K., Tsakirakis N., Malandrakis P., Vitsos P., Metousis A., Orologas-Stavrou N., Ntanasis-Stathopoulos I., Kanellias N., Eleutherakis-Papaiakovou E., Pothos P. (2020). Deep Phenotyping Reveals Distinct Immune Signatures Correlating with Prognostication, Treatment Responses, and MRD Status in Multiple Myeloma. Cancers.

[16] Puig N., Paiva B., Lasa M., Burgos L., Perez J.J., Merino J., Moreno C., Vidriales M.B., Toboso D.G., Cedena M.T. (2019). Flow cytometry for fast screening and automated risk assessment in systemic light-chain amyloidosis. Leukemia.

[17] Kumar S., Paiva B., Anderson K.C., Durie B., Landgren O., Moreau P., Munshi N., Lonial S., Bladé J., Mateos M.V. (2016). International Myeloma Working Group consensus criteria for response and minimal residual disease assessment in multiple myeloma. Lancet Oncol..

[18] Sonneveld P., Broijl A. (2016). Treatment of relapsed and refractory multiple myeloma. Haematologica.

[19] Krzywdzińska A., Puła B., Czyż A., Krzymieniewska B., Kiernicka-Parulska J., Mierzwa A., Szymczak D., Milanowska A., Kiraga A., Kwiecień I. (2021). Harmonization of flow cytometric minimal residual disease assessment in multiple myeloma in centers of Polish Myeloma Consortium. Diagnostics.

[20] Stetler-Stevenson M., Paiva B., Stoolman L., Lin P., Jorgensen J.L., Orfao A., Van Dongen J., Rawstron A.C. (2015). Consensus guidelines for myeloma minimal residual disease sample staining and data acquisition. Cytom. B Clin. Cytom..

[21] Mendonça de Pontes R., Flores-Montero J., Sanoja-Flores L., Puig N., Pessoa de Magalhães R.J., Corral-Mateos A., Salgado A.B., García-Sánchez O., Pérez-Morán J., Mateos M.V. (2021). B-Cell regeneration profile and minimal residual disease status in bone marrow of treated multiple myeloma patients. Cancers.

[22] Guillerey C., Nakamura K., Vuckovic S., Hill G.R., Smyth M.J. (2016). Immune responses in multiple myeloma: Role of the natural immune surveillance and potential of immunotherapies. Cell Mol. Life Sci..

[23] Zheng Y., Cai Z., Wang S., Zhang X., Qian J., Hong S., Li H., Wang M., Yang J., Yi Q. (2009). Macrophages are an abundant component of myeloma microenvironment and protect myeloma cells from chemotherapy drug-induced apoptosis. Blood.

[24] Terpos E., Kostopoulos I.V., Kastritis E., Ntanasis-Stathopoulos I., Migkou M., Rousakis P., Argyriou A.T., Kanellias N., Fotiou D., Eleutherakis-Papaiakovou E. (2019). Impact of minimal residual disease detection by Next-Generation Flow Cytometry in multiple myeloma patients with sustained complete remission after frontline therapy. HemaSphere.

[25] Mohan M., Kendrick S., Szabo A., Yarlagadda N., Atwal D., Pandey Y., Roy A., Parikh R., Lopez J., Thanendrarajan S. (2022). Clinical implications of loss of bone marrow minimal residual disease negativity in multiple myeloma. Blood Adv..

[26] Arteche-López A., Kreutzman A., Alegre A., Sanz Martín P., Aguado B., González-Pardo M., Espiño M., Villar L.M., García Belmonte D., de la Cámara R. (2017). Multiple myeloma patients in long-term complete response after autologous stem cell transplantation express a particular immune signature with potential prognostic implication. Bone Marrow Transplant..

[27] Perez C., Botta C., Zabaleta A., Puig N., Cedena M.T., Goicoechea I., Alameda D., San José-Eneriz E., Merino J., Rodríguez-Otero P. (2020). Immunogenomic identification and characterization of granulocytic myeloid-derived suppressor cells in multiple myeloma. Blood.

[28] San Miguel J.F., Almeida J., Mateo G., Blade J., Lopez-Berges C., Caballero D., Hernandez J., Moro M.J., Fernandez-Calvo J., Diaz-Mediavilla J. (2002). Immunophenotypic evaluation of the plasma cell compartment in multiple myeloma: A tool for comparing the efficacy of different treatment strategies and predicting outcome. Blood.

[29] Pessoa de Magalhães R.J., Vidriales M.B., Paiva B., Fernandez-Gimenez C., García-Sanz R., Mateos M.V., Gutierrez N.C., Lecrevisse Q., Blanco J.F., Hernández J. (2013). Analysis of the immune system of multiple myeloma patients achieving long-term disease control by multidimensional flow cytometry. Haematologica.

[30] Botta C., Pérez Ruiz C., Goicoechea I., Puig N., Cedena M.T., Cordon L., Zabaleta A., Burgos L., Maia C., Rodríguez S. (2019). Single-cell characterization of the multiple myeloma (MM) immune microenvironment identifies CD27-negative T cells as potential source of tumor-reactive lymphocytes. Blood.

[31] Koehne G., Zeller W., Stockschlaeder M., Zander A.R. (1997). Phenotype of lymphocyte subsets after autologous peripheral blood stem cell transplantation. Bone Marrow Transplant..

[32] Dosani T., Carlsten M., Maric I., Landgren O. (2015). The cellular immune system in myelomagenesis: NK cells and T cells in the development of myeloma [corrected] and their uses in immunotherapies. Blood Cancer J..

[33] Jurisic V., Srdic T., Konjevic G., Markovic O., Colovic M. (2007). Clinical stage-depending decrease of NK cell activity in multiple myeloma patients. Med. Oncol..

[34] García-Ortiz A., Rodríguez-García Y., Encinas J., Maroto-Martín E., Castellano E., Teixidó J., Martínez-López J. (2021). The role of tumor microenvironment in multiple myeloma development and progression. Cancers.

[35] Hayashi T., Hideshima T., Akiyama M., Podar K., Yasui H., Raje N., Kumar S., Chauhan D., Treon S.P., Richardson P. (2005). Molecular mechanisms whereby immunomodulatory drugs activate natural killer cells: Clinical application. Br. J. Haematol..

[36] Giles A.J., Hutchinson M.-K., Sonnemann H.M., Jung J., Fecci P.E., Ratnam N.M., Zhang W., Song H., Bailey R., Davis D. (2018). Dexamethasone-induced immunosuppression: Mechanisms and implications for immunotherapy. J. Immunother. Cancer..

[37] Holstein S.A., McCarthy P.L. (2017). Immunomodulatory drugs in multiple myeloma: Mechanisms of action and clinical experience. Drugs.

[38] Firer M.A., Shapira M.Y., Luboshits G. (2021). The impact of induction regimes on immune responses in patients with multiple myeloma. Cancers.

[39] Lopes R., Caetano J., Ferreira B., Barahona F., Carneiro E.A., João C. (2021). The immune microenvironment in multiple myeloma: Friend or foe?. Cancers.

[40] Visram A., Dasari S., Anderson E., Kumar S., Kourelis T.V. (2021). Relapsed multiple myeloma demonstrates distinct patterns of immune microenvironment and malignant cell-mediated immunosuppression. Blood Cancer J..

[41] Hadjiaggelidou C., Katodritou E. (2021). Regulatory T-cells and cultiple cyeloma: Implications in tumor immune biology and treatment. J. Clin. Med..

[42] Verma A., Mathur R., Farooque A., Kaul V., Seema Gupta S., Dwarakanath B.S. (2019). T-regulatory cells in tumor progression and therapy. Cancer Manag. Res..

[43] Cencini E., Fabbri A., Sicuranza A., Gozzetti A., Bocchia M. (2021). The role of tumor-associated macrophages in hematologic malignancies. Cancers.

[44] Mina R., Belotti A., Petrucci M.T., Zambello R., Capra A., Di Lullo G., Ronconi S., Pescosta N., Grasso M., Monaco F. (2020). Bortezomib-dexamethasone as maintenance therapy or early retreatment at biochemical relapse versus observation in relapsed/refractory multiple myeloma patients: A randomized phase II study. Blood Cancer J..

